# Associations between food-related concerns, food security status, and food support use: a secondary analysis of the Food and You 2: Wave 6 dataset

**DOI:** 10.1017/jns.2025.10065

**Published:** 2026-01-09

**Authors:** Natalie Taylor, Paul Christiansen, Beth Armstrong, Emma Boyland, Charlotte A. Hardman

**Affiliations:** 1 Department of Psychology, University of Liverpool, Liverpool, UK; 2 Department of Geography and Planning, University of Sheffield, Sheffield, UK

**Keywords:** Food prices, Food quality, Food security, Food waste, Healthy eating

## Abstract

Household food insecurity has previously been associated with psychological distress, and subsequently, poorer diet quality. Further understanding of this relationship is required to improve nutritional outcomes, with food-related concerns suggested as one potential mechanism. Therefore, the current pre-registered (https://osf.io/zd3ak) study conducted cross-sectional secondary analyses of Wave 6 (October 2022–January 2023) of the Food and You 2 survey administered in adults aged 16 years and over across England, Wales, and Northern Ireland (*N* = 2315), to explore the differential prevalence of food-related concerns in people experiencing food insecurity. Exploratory analyses also identified characteristics of food support users (food bank or social supermarket; *N* = 467) and quantified associations between food support use and the same food-related concerns. People experiencing marginal (OR = 1.43, *p* = 0.02) and low food security (OR = 1.51, *p* = 0.02) (relative to high food security) were significantly more concerned about food prices, but this association was not seen in people experiencing very low food security. Both food bank and social supermarket use were predicted by very low food security (food bank OR = 6.05, *p* < 0.001; social supermarket OR = 2.40, *p* = 0.02) and having a long-term health condition (food bank OR = 3.91, *p* = 0.00; social supermarket OR = 3.17, *p* = 0.00). Food bank users were less concerned about healthy eating (OR = 0.33, *p* = 0.00) whereas social supermarket users were less concerned about food prices (relative to non-users) (OR = 0.40, *p* = 0.01). Food-related concerns, particularly regarding food prices, are differentially associated with food security status and food support use. Findings could support specific interventions to promote better diet quality and improve health and wellbeing in populations experiencing food insecurity.

## Introduction

### Background

Globally, food insecurity is a significant issue that affects over 295 million people.^(1)^ On a national scale, in the wake of the current cost-of-living crisis, now, more than ever, the gravity of the problem of food insecurity in the UK demands serious public health attention.^(2,3)^ Recent data from the Foods Standards Agency (FSA) in the UK shows that 25% of households responding to their national survey, Food and You 2, are now experiencing food insecurity, compared to 16% in 2020,^(4)^ likely due to a sharp rise in living costs, without a respective increase in household incomes.^(5,6)^

Food insecurity is defined by Radimer, Olson^(7)^ as uncertain access to a sufficient quantity of safe and nutritious food to satisfy household needs for health and wellbeing, and can be experienced across a spectrum of severity based upon difficulties or limitations in accessing food and the resulting impact upon dietary quality and variety, and food intake.^(8)^ Across the literature, the significant impacts of food insecurity on health and wellbeing have been well-documented. Concerning physical health, food insecurity has been linked to the proliferation and exacerbation of a number of non-communicable diseases including hypertension, diabetes and obesity.^(9–14)^ Meanwhile, the relationship between food insecurity and mental health outcomes including depression, anxiety and stress is well-evidenced^(9,12,15,16)^ and has been hypothesised to mediate the association between food insecurity and physical health outcomes.^(9,17,18)^ For example, a recent study found that household food insecurity was indirectly associated with diet quality through distress (measured through depression, anxiety, and stress).^(19)^ Similarly, distress has also been found to mediate the association between food insecurity and both eating to cope and higher body mass index (BMI).^(20)^ However, despite its clear impacts upon diet quality and health, the mechanism underpinning the association between food insecurity and distress is not well understood. Further understanding this relationship may contribute towards identifying potential points of intervention to improve nutritional outcomes for people experiencing food insecurity.

One potential mechanism may be the manifestation of concern and worry over food, associated with food insecurity.^(21–23)^ For example, in a study conducted in Canada during the Covid-19 pandemic, worry about affording food was significantly associated with higher odds of reporting symptoms of anxiety and depression.^(24)^ However, according to the definition set out by Radimer, Olson,^(7)^ the experience of food insecurity engenders concern not only around the affordability of food, but also regarding nutritional and actual quality, quantity, and uncertainty surrounding access to food both in the present and the future.^(25–27)^ Additionally, widely used measures of food insecurity, such as the USDA 10-item Adult Food Security Survey Module (AFSSM) even reference worry in their items (e.g. ‘I worried whether my food would run out before I got money to buy more’),^(8)^ indicating its significance within the food insecurity experience. Meanwhile, food insecurity has also been linked to specific food management strategies (or coping strategies) including the strict use of resourceful cooking and making food last longer to reduce household food waste.^(3,16,20,25,28–31)^ The pressure of adhering to these strategies might also manifest in higher levels of worry and concern about food waste than would be seen in food secure populations,^(30)^ contributing towards negative psychological impacts amongst individuals experiencing food insecurity.^(25,32,33)^ For people accessing food support services such as food banks (synonymous with food pantries in the USA) and social supermarkets (otherwise known as food clubs, hubs or community food stores, offering a shop-like environment in which individuals can purchase food and other household essentials for highly reduced prices), the level of food-related concerns is likely to be more substantial, due to the presumably higher severity of food insecurity experienced in this population. Additionally, healthy foods have been shown to be over twice as expensive per calorie than their less healthy counterparts,^(34,35)^ meaning that concerns about food prices and being able to eat healthily may now affect not only people experiencing food insecurity, but those also considered food secure, potentially driving higher distress and thus poorer nutritional outcomes in the general population too. For example, data from the most recent FSA Consumer Insights Tracker (January–March 2025) indicated that the top food concern reported by households across the UK was food prices, having been consistently reported as such across monthly iterations of the survey for over a year.^(36)^

The presence and lived experience of these food-related concerns in people experiencing food insecurity and using food support services has been documented previously within qualitative research across high income countries.^(16,30,37–41)^ Yet, thus far, this area is underexplored quantitatively, and to our knowledge, an exploration of the differential ways that food-related concerns are experienced across food security statuses and food support types has not yet been conducted. Exploring food-related concerns across differing levels of food security severity (high, marginal, low, and very low food security) might help to define areas of specific worry and contribute towards developing interventions to address these concerns in order to minimise the psychological impact of food insecurity more effectively, in turn promoting more healthy food choices and improved health in people experiencing food insecurity and beyond. Similarly, understanding how use of food support (e.g. use of food banks and social supermarkets) is associated with food-related concerns might elucidate the most psychologically supportive services and highlight where intervention efforts might be best focused.

### Aims of the study

Using data from the Food Standards Agency (FSA) Food and You 2: Wave 6 survey, this study primarily aimed to quantify the associations between different levels of food security severity and four food-related concerns; (1) food prices, (2) food waste, (3) food quality, and (4) being able to eat healthily (Research Question (RQ) 1). These food-related concerns were chosen as they were all accessible within the Food and You 2 dataset and considered to be important concepts relating to the experience and definition of food insecurity. It was hypothesised that a lower food security status (e.g. very low, low, or marginal food security) would predict a higher likelihood of reporting concerns in these four areas, relative to high food security status. Exploratory analyses were also conducted to explore the (i) characteristics of those using food banks and social supermarkets compared to non-users (RQ2), and (ii) associations between the four food-related concerns and food bank or social supermarket use (RQ3). Exploration of the characteristics of food bank clients may identify certain demographics who can be targeted with tailored support to improve access to this type of food support, and thus the nutritional quality of their diet. Additionally, the characteristics of social supermarket users are little explored, with this model of food support being relatively new.^(42)^ Finally, for those experiencing more severe forms of food insecurity, the associations between accessing different types of food support and food-related concerns are also not well understood.

## Methods

### Participants and study design

The present pre-registered (https://osf.io/zd3ak) study used data from Food and You 2: Wave 6, an Official Statistic survey administered across England, Wales, and Northern Ireland by the FSA. Food and You 2 consists of a biannual cross-sectional survey measuring self-reported knowledge, attitudes, and behaviour relating to food. Fieldwork for Food and You 2: Wave 6 was conducted between 12 October 2022 and 10 January 2023 for the FSA by Ipsos, an external multinational market research and consultancy firm, with the data collected undergoing secondary analysis in the present study. The survey was completed by 5991 participants aged over 16 years, residing in 4217 households across England, Wales, and Northern Ireland. A sample of postcodes across England, Wales, and Northern Ireland were selected using stratified random probability sampling. Participants were sent a postal invitation to complete the push-to-web survey, enabling respondents to complete an online or postal version of the survey. To reduce participant burden and encourage participation, the postal version of the survey contained fewer questions than the online version. Weights were applied to the data to make the respondent sample nationally representative and to account for differences in survey length across postal and online versions.^(43)^ Further details of the sampling design, method, weighting, and survey procedure can be found in the Technical report issued by the FSA.^(43)^ The full survey can also be accessed online: (https://www.food.gov.uk/sites/default/files/media/document/FY2%20Wave%206%20Online%20questionnaire_V1_Public.pdf).

### Measures

All of the following measures were used within the Food and You 2 Wave 6 survey, and the data collected have been used for secondary data analysis in the present study. All variables were entered into the models quantitatively.

#### Food security status

Food security status was measured using the United States Department for Agriculture (USDA) 10-item AFSSM.^(8)^ The USDA 10-item AFSSM is widely used across national government- and third sector-commissioned surveys and represents a well validated measure of food insecurity within the UK.^(44)^ The 10 items encapsulate difficulties in affording sufficient food for household needs (e.g. ‘The food that I bought just didn’t last and I didn’t have money to get more’), worry about future access to food (e.g. ‘I worried whether my food would run out before I got money to buy more’) and subsequent food and eating behaviours including eating less, skipping meals, and going hungry (e.g. ‘In the last 12 months, did you ever cut the size of your meals or skip meals because there wasn’t enough money for food?’). Affirmative responses to these items are summed into a raw score out of 10 and from the raw score respondents are classified as experiencing high (raw score = 0), marginal (raw score = 1–2), low (raw score = 3–5) or very low (raw score = 6–10) food security.^(8)^ High food security is defined as no reported difficulties or limitations in accessing food; marginal food security is defined as showing some concern or anxiety over uncertain access to food but no tangible change in diet or food intake; low food security is defined as consuming a diet of reduced quality, variety and adherence to dietary preferences, but that is not reduced in quantity; and very low food security is defined as a reduction in both quality and quantity, and a consequent disruption of eating patterns.^(8,45)^ McDonald’s omega was above the acceptable value of 0.7^(46)^ for the USDA AFSSM (ⲱ_t_ = 0.91), showing good internal reliability in this sample.

#### Food bank and social supermarket use

Food and You 2 survey participants were asked ‘In the last 12 months, have you, or anyone else in your household received a free parcel of food from a food bank or other emergency food provider?’ and ‘In the last 12 months, have you, or anyone else in your household, used a social supermarket (also known as a food club/hub or community pantry)?’. Possible responses were ‘Yes’, ‘No’, and ‘Prefer not to say’. Regarding social supermarket use, participants also had the option ‘I had not heard of a social supermarket, food club/hub or community pantry before today’.

#### Food-related concerns

Food and You 2 survey participants were presented with a list of 22 items covering possible food-related concerns including but not limited to food safety, environment, nutrition, and food packaging and asked to indicate whether they had concerns about them (‘Do you have any concerns about the following?’). If selected, the food-related concern would be coded as ‘Yes’ and if not selected, the food-related concern was coded as ‘No’. The four items of interest in this study were ‘food prices’, ‘food waste’, ‘food quality’, and ‘being able to eat healthily’, due to their relevance and relationship to the definition of food insecurity. Due to food-related concern variables being binary, tetrachoric correlation coefficients were computed to understand correlations between concerns. Concerns about being able to eat healthily and food quality were considered to be strongly correlated (r_tet_ = 0.51), while food waste concerns were moderately correlated to food quality concerns (r_tet_ = 0.35) and being able to eat healthily (r_tet_ = 0.41) (based on standard effect sizes for Pearson correlation due to lack of consensus on tetrachoric correlation effect sizes).^(47)^ All other correlations were considered to be weak (see Supplementary Material 1).

#### Demographic covariates

Several socioeconomic and demographic variables (gender, age, country, urban/rural classification, employment status, ethnicity, presence of long-term health condition, total household size, and total household income) were used to characterise the population of food bank and social supermarket users compared to non-users (RQ2) and to act as covariates in the analyses (see Table 1). These variables represent a selected sample from the Food and You 2 survey items which were deemed most relevant to the research context, known to be linked to the prevalence of food insecurity and thus having the potential to confound the analysis. Gender (male; female), ethnicity (White; other ethnic group), presence of a long-term health condition (has long-term health condition; doesn’t have long-term health condition), and urban/rural classification (urban; rural) were considered binary variables throughout the analysis. Country, employment status, age, total household size, and total annual household income were all considered categorical variables, unless otherwise stated (see section *Food-related concerns and food support (RQ2, RQ3)*). All categorical variables were assigned a number (e.g. for the gender variable, male was assigned 1 and female was assigned 2) in order for the data to be analysed quantitatively.

**Table 1.** Summary of socioeconomic and demographic variables used for descriptive statistics and acting as covariates, along with their corresponding categories

Variable	Categories
Gender	Male
	Female
Age*	16–24
	25–34
	35–44
	45–54
	55–64
	65–79
	80+
Country	England
	Wales
	Northern Ireland
Urban/rural classification	Urban
	Rural
Employment status	Working
	Unemployed/not working
	Retired
Ethnicity	White
	Non-White
Presence of a long-term health condition	Has a long-term health condition
	Doesn’t have a long-term health condition
Total household size*	1
	2
	3
	4
	5+
Total annual household income*	<£19,000
	£19,000–£31,999
	£32,000–£63,999
	£64,000–£95,999
	>£96,000

* Age, total household size and total annual household income were considered categorical variables unless otherwise stated.

### Data analysis

The dataset was accessed from the UK Data Service website. Responses of ‘Not stated’, ‘Not applicable’, ‘Don’t know’, and ‘Prefer not to say’ to each variable were removed from the data. The data file (*N* = 2315) was then weighted (see Food and You 2: Wave 6 Technical report^(43)^) in SPSS version 29. The SPSS data files were then read into RStudio version 4.3.1 for the analysis. All data files and R scripts are available on the Open Science Framework (https://osf.io/72dsz). Outcomes were considered statistically significant if *p* < 0.05.

#### Food security status and food-related concerns (RQ1)

To determine the extent to which food security status could predict food-related concerns, a two-step logistic regression model was conducted for each food-related concern in turn. In the first step, the respective food-related concern was the dependent variable (binary, categorical variable coded as ‘Yes’ = 1 and ‘No’ = 0), and the covariates listed in Table 1 were entered as predictors. All models were significant for step 1 (see Supplementary Material 2). In the second step, food security status was added to the model (independent variable). Food security status was considered a four-level categorical variable in the analysis, coded so that *higher scores* indicated *lower food security* and therefore *higher food insecurity* (high food security = 1; marginal food security = 2; low food security = 3; very low food security = 4). The categories ‘very low food security’, ‘low food security’, and ‘marginal food security’ were all compared to the reference category ‘high food security’. An analysis of deviance assessed whether food security status improved the predictive utility of the previous model. Odds ratios and 95% confidence intervals are reported from the final model.

#### Food-related concerns and food support (RQ2, RQ3)

For the exploratory research questions, the sample was reduced to only participants who were categorised as experiencing food insecurity (very low or low food security; *N* = 467), as it was considered that people living in high or marginal food security would not be regularly receiving food support from food support services due to their ‘last resort’ nature, more likely implementing coping strategies to cope with food shortage.^(16,26,31,48)^ To characterise the populations of participants using food banks and social supermarkets compared to those that do not (RQ2), logistic regressions were conducted with the binary categorical variables ‘food bank use’ or ‘social supermarket use’ as the dependent variables (recoded as ‘Yes’ = 1 and ‘No’ = 0 for clarity during data analysis and interpretation). The socioeconomic and demographic variables in Table 1 were used as covariates in the model for RQ2. Food security status (including low and very low food security status) was also added as a covariate for analysis under RQ2, in order to explore differences in self-reported food support use between people in low and very low food security. Within the analysis for RQ2, food security status, gender, age, long-term health condition, urban/rural classification, and ethnicity were considered binary categorical variables; country and employment were considered three-level categorical variables; age, total household size, and total annual household income were treated as continuous variables, as there was no clear comparator (see Supplementary Material 3).

To determine the extent to which self-reported receipt of food support from a food bank or social supermarket could predict food-related concerns (RQ3), a similar two-step analysis strategy was implemented as for RQ1 (see section *Food security status and food-related concerns (RQ1)*). A logistic regression was conducted with each food-related concern in turn as the dependent variable and covariates only as predictors (excluding food security status, as in RQ1). All models were statistically significant for step 1 (see Supplementary Material 4). Food bank or social supermarket use then was added as the independent variable to each model in a second step. Analysis of deviance was again conducted to assess whether the predictive utility of the model could be improved by adding food bank or social supermarket use, compared to the covariate only model.

## Results

### Food security status and food-related concerns (RQ1)

#### Descriptive statistics

The sample characteristics (*N* = 2315) can be found in Table 2. Food security status indicated that 67.6% were in high food security and 12.3% were experiencing marginal food security, while 10.2% and 10% respectively were in low and very low food security. Despite this, in terms of receipt of food support, only 2.6% of the whole sample reported using a food bank and just 2.4% reported using a social supermarket. Additionally, descriptive frequencies of food-related concerns across the whole sample and stratified by food security status, food bank use, and social supermarket use are presented in Table 3.

**Table 2.** Descriptive statistics of the sample characteristics (*N* = 2315)

Measure	n(%)	Food security status				Food bank use		Social supermarket use	
		H	M	L	VL	Y	N	Y	N
		1564 (67.6)	284 (12.3)	236 (10.2)	231 (10.0)	60 (2.6)	2255 (97.6)	56 (2.4)	2259 (97.6)
Ethnicity									
White	2196 (94.9)	1505 (96.2)	263 (92.6)	214 (90.7)	214 (92.6)	54 (90.0)	2142 (95.0)	47 (83.9)	2149 (95.1)
Other ethnic group	119 (5.1)	59 (3.8)	21 (7.4)	22 (9.3)	17 (7.4)	6 (10.0)	113 (5.0)	9 (16.1)	110 (4.9)
Country									
England	1081 (46.7)	767 (49.0)	114 (40.1)	103 (43.6)	97 (42.0)	26 (43.3)	1055 (46.8)	31 (55.4)	1050 (46.5)
Wales	622 (26.9)	412 (26.3)	89 (31.3)	60 (25.4)	61 (26.4)	17 (28.3)	605 (26.8)	10 (17.9)	612 (27.1)
Northern Ireland	612 (26.4)	385 (24.6)	81 (28.5)	73 (30.9)	73 (31.6)	17 (28.3)	595 (26.4)	15 (26.8)	597 (26.4)
Gender									
Male	1012 (43.7)	741 (48.4)	110 (39.7)	86 (37.4)	75 (32.5)	21 (35.0)	991 (44.9)	16 (28.6)	996 (44.1)
Female	1303 (56.3)	823 (52.6)	174 (61.3)	150 (63.6)	156 (67.5)	39 (65.0)	1264 (56.1)	40 (71.4)	1263 (55.9)
Age band									
16–24	85 (3.7)	37 (2.4)	15 (5.3)	17 (7.2)	16 (6.9)	6 (10.0)	79 (3.5)	8 (14.3)	77 (3.4)
25–34	322 (13.9)	137 (8.8)	57 (20.1)	53 (22.5)	75 (32.5)	17 (28.3)	305 (13.5)	15 (26.8)	307 (13.6)
35–44	403 (17.4)	212 (13.6)	61 (21.5)	58 (24.6)	72 (31.2)	16 (26.7)	387 (17.2)	15 (26.8)	388 (17.2)
45–54	433 (18.7)	311 (19.9)	51 (18.0)	39 (16.5)	32 (13.9)	11 (18.3)	422 (18.7)	8 (14.3)	425 (18.8)
55–64	503 (21.7)	399 (25.5)	46 (16.2)	39 (16.5)	19 (8.2)	6 (10.0)	497 (22.0)	6 (10.7)	497 (22.0)
65–79	508 (21.9)	416 (26.6)	48 (16.9)	28 (11.9)	16 (6.9)	4 (6.7)	504 (22.4)	4 (7.1)	504 (22.3)
80+	61 (2.6)	52 (3.3)	6 (2.1)	2 (0.8)	1 (0.4)	0 (0)	61 (2.7)	0 (0.0)	61 (2.7)
Urban/rural classification									
Urban	1617 (69.8)	1065 (68.1)	206 (72.5)	172 (72.9)	174 (76.3)	42 (70.0)	1575 (69.8)	43 (76.8)	1574 (69.7)
Rural	698 (30.2)	499 (31.9)	78 (27.5)	64 (27.1)	57 (24.7)	18 (30.0)	680 (30.2)	13 (23.2)	685 (30.3)
Food security status									
High food security	1564 (67.6)	1564 (100)	0 (0)	0 (0)	0 (0)	4 (6.7)	1560 (69.2)	8 (14.3)	1556 (68.9)
Marginal food security	284 (12.3)	0 (0)	284 (100)	0 (0)	0 (0)	7 (11.7)	277 (12.3)	8 (14.3)	276 (12.2)
Low food security	236 (10.2)	0 (0)	0 (0)	236 (100)	0 (0)	6 (10.0)	230 (10.2)	9 (16.1)	227 (10.0)
Very low food security	231 (10.0)	0 (0)	0 (0)	0 (0)	231 (100)	43 (71.7)	188 (8.3)	31 (55.4)	200 (8.9)
Employment status									
Working	1447 (62.5)	953 (60.9)	181 (63.7)	165 (69.9)	148 (64.1)	25 (41.7)	1422 (63.1)	30 (53.6)	1417 (62.7)
Unemployed/not working	246 (10.6)	100 (6.4)	37 (13.0)	38 (16.1)	71 (30.7)	31 (51.7)	215 (9.5)	23 (41.1)	223 (9.9)
Retired	622 (26.9)	511 (32.7)	66 (23.2)	33 (14.0)	12 (5.2)	4 (6.7)	618 (27.4)	3 (5.4)	619 (27.4)
Total annual household income									
<£19,000	520 (22.5)	231 (14.8)	77 (27.1)	89 (37.7)	123 (53.2)	40 (66.7)	480 (21.3)	30 (53.6)	490 (21.7)
£19,000–£31,999	582 (25.1)	350 (22.4)	86 (30.3)	75 (31.8)	71 (30.7)	15 (25.0)	567 (25.1)	17 (30.4)	565 (25.0)
£32,000–£63,999	792 (34.2)	606 (38.7)	94 (33.1)	61 (25.8)	31 (13.4)	5 (8.3)	787 (34.9)	6 (10.7)	786 (34.8)
£64,000–£95,999	282 (12.2)	247 (15.8)	22 (7.7)	7 (3.0)	6 (2.6)	0 (0.0)	282 (12.5)	3 (5.4)	279 (12.4)
>£96,000	139 (6.0)	130 (8.3)	5 (1.8)	4 (1.7)	0 (0.0)	0 (0.0)	139 (6.2)	0 (0.0)	139 (6.2)
Total household size									
1	301 (13.0)	200 (12.8)	38 (13.4)	32 (13.6)	31 (13.4)	10 (16.7)	291 (12.9)	7 (12.5)	294 (13.0)
2	1081 (46.7)	834 (53.3)	110 (38.7)	83 (35.2)	54 (23.4)	14 (23.3)	1067 (47.3)	14 (25.0)	1067 (47.2)
3	366 (15.8)	214 (13.7)	54 (19.0)	44 (18.6)	54 (23.4)	14 (23.3)	352 (15.6)	11 (19.6)	355 (15.7)
4	378 (16.3)	218 (13.9)	60 (21.1)	45 (19.1)	55 (23.8)	11 (18.3)	367 (16.3)	15 (26.8)	363 (16.1)
5+	189 (8.2)	98 (6.3)	22 (7.7)	32 (13.6)	37 (16.0)	11 (18.3)	178 (7.9)	9 (16.1)	180 (8.0)
Long-term health condition									
Has a long-term health condition	716 (30.9)	427 (27.3)	92 (32.4)	83 (35.2)	114 (49.4)	39 (65.0)	677 (30.0)	35 (62.5)	681 (30.1)
Doesn’t have a long-term health condition	1599 (69.1)	1137 (72.7)	192 (67.6)	153 (64.8)	117 (50.6)	21 (35.0)	1578 (70.0)	21 (37.5)	1578 (69.9)
Food bank use									
Yes	60 (2.6)	4 (0.3)	7 (2.5)	6 (2.5)	43 (18.6)	60 (100)	0 (0.0)	27 (48.2)	33 (1.5)
No	2255 (97.4)	1560 (99.7)	277 (97.5)	230 (97.5)	188 (81.4)	0 (0.0)	2255 (100)	29 (51.8)	2226 (98.5)
Social supermarket use									
Yes	56 (2.4)	8 (0.5)	8 (2.8)	9 (3.8)	31 (13.6)	27 (45.0)	29 (1.3)	56 (100)	0 (0.0)
No	2259 (97.6)	1556 (99.5)	276 (97.2)	227 (96.2)	200 (86.6)	33 (55.0)	2226 (98.7)	0 (0.0)	2259 (100)

H, high food security; M, marginal food security; L, low food security; VL, very low food security; Y, Yes; N, No.

Chi-squared tests were conducted to explore bivariate associations between food security status and the following covariates (with all associations being statistically significant at *p* < 0.05): ethnicity *Ⲭ* ^2^(3) = 19.76, *p* < 0.001, *V* = 0.09; country *Ⲭ* ^2^(6) = 15.06, *p* = 0.020, *V* = 0.06; gender *Ⲭ* ^2^(3) = 28.35, *p* < 0.001, *V* = 0.11; age band *Ⲭ* ^2^(18) = 284.27, *p* < 0.001, *V* = 0.20; urban/rural classification *Ⲭ* ^2^(3) = 7.58, *p* < 0.056, *V* = 0.06; employment status *Ⲭ* ^2^(6) = 201.3, *p* < 0.001, *V* = 0.21; total annual household income *Ⲭ* ^2^(12) = 322.90, *p* < 0.001, *V* = 0.22; total household size *Ⲭ* ^2^(12) = 119.55, *p* < 0.001, *V* = 0.13; long-term health condition *Ⲭ* ^2^(3) = 48.6, *p* < 0.001, *V* = 0.14; food bank use *Ⲭ* ^2^(3) = 268.74, *p* < 0.001, *V* = 0.34; social supermarket use *Ⲭ* ^2^(3) = 144.67, *p* < 0.001, *V* = 0.25.

**Table 3.** Descriptive frequencies of food-related concerns across the whole sample and stratified by food security status, food bank use and social supermarket use

			Food security status				Food bank use		Social supermarket use	
Outcome		n(%)	H	M	L	VL	Yes	No	Yes	No
			1564 (67.6)	284 (12.3)	236 (10.2)	231 (10.0)	60 (2.6)	2255 (97.6)	56 (2.4)	2259 (97.6)
Food prices	**Yes**	1556 (67.2)	1013 (64.8)	207 (72.9)	176 (74.6)	160 (69.3)	38 (63.3)	1518 (67.3)	34 (60.7)	1522 (67.4)
	**No**	759 (32.8)	551 (35.2)	77 (27.1)	60 (25.4)	71 (30.7)	22 (36.7)	737 (32.7)	22 (39.3)	737 (32.6)
Food waste	**Yes**	1483 (64.1)	1088 (69.6)	162 (57.0)	122 (51.7)	111 (48.1)	31 (51.7)	1452 (64.4)	29 (51.8)	1454 (64.4)
	**No**	832 (35.9)	476 (30.4)	122 (43.0)	114 (48.3)	120 (51.9)	29 (48.3)	803 (35.6)	27 (48.2)	805 (35.6)
Food quality	**Yes**	1197 (51.7)	1044 (66.8)	174 (61.3)	140 (59.3)	114 (49.4)	28 (46.7)	1444 (64.0)	25 (44.6)	1447 (64.1)
	**No**	1118 (48.3)	520 (33.2)	110 (38.7)	96 (40.7)	117 (50.6)	32 (53.3)	811 (36.0)	31 (55.4)	812 (35.9)
Being able to eat healthily	**Yes**	1472 (63.6)	794 (50.8)	139 (48.9)	103 (43.6)	82 (35.5)	13 (21.7)	1105 (49.0)	20 (35.7)	1098 (48.6)
	**No**	843 (36.4)	770 (49.2)	145 (51.1)	133 (56.4)	149 (64.5)	47 (78.3)	1150 (51.0)	36 (64.3)	1161 (51.4)

#### Logistic regressions 1–4: Food security status and food-related concerns

Variance inflation factors (VIFs) were calculated for each step in the two-step model. All VIFs < 2 for the covariates in the models in RQ1, indicating minimal multicollinearity between covariates.^(49)^ Upon adding food security status into each model, the model fit was significantly improved for both food price concerns (*p* = 0.02) and food waste concerns (*p* = 0.01) but was not significantly improved for food quality concerns and concerns about being able to eat healthily (see Table 4).

**Table 4.** Change in model fit statistics for regressions with food-related concerns against covariates and food insecurity status

Food-related concern	ΔⲬ^2^	*Df*	*p*	Δ(McFadden *R* ^2^)	Δ(Classification rate)	ΔAIC
Regression 1: Food prices	9.95	3	0.02*	0	34.5%	−4
Regression 2: Food waste	11.52	3	0.01*	0	0.5%	−5.5
Regression 3: Food quality	6.79	3	0.08	0.01	0.2%	−0.8
Regression 4: Being able to eat healthily	4.17	3	0.24	0	0.4%	1.8

*p* < 0.05*, *p* < 0.001**.

RQ1: **Dependent variable:** Food-related concern; **Independent variable:** Food security status; **Covariates:** ethnicity, country, gender, age band, urban/rural classification, employment status, total annual household income, total household size, long-term health condition.

For Regression 1: Food prices, the only covariates that were significant were age, total household size, country, and employment status. All other covariates were non-significant.

For Regression 2: Food waste, the only covariates that were significant were gender, age, total household size, and total household income. All other covariates were non-significant.

For Regression 3: Food quality, the only covariates that were significant were age and presence of a long-term health condition. All other covariates were non-significant.

For Regression 4: Being able to eat healthily, the only covariates that were significant were age and total household income. All other covariates were non-significant.

**Figure 1.**
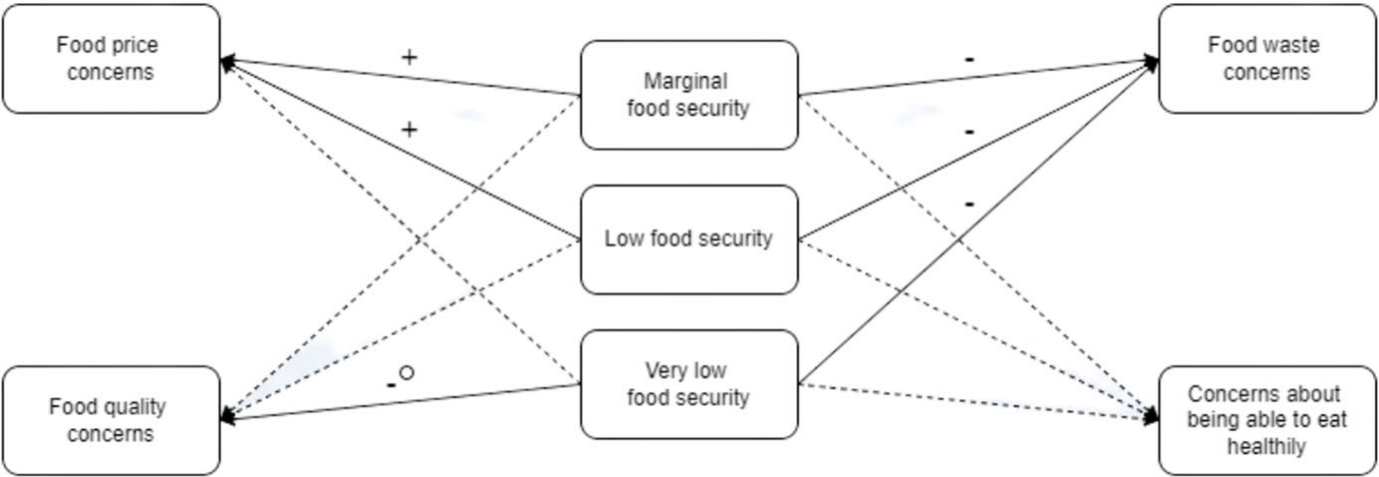
A visual overview of the associations between food security status and food-related concerns. Solid arrows denote significant associations and dashed arrows denote non-significant associations. Positive associations are represented by a ‘+’ and negative associations are represented by a ‘−’ above their associated arrow. The association between very low food security status and food quality concerns was not found to be independent of the covariates.

Food security status showed some associations to food-related concerns (see Supplementary Material 5 and Figure 1). Food security status was associated with food price concerns (Regression 1). Participants experiencing marginal (OR = 1.43(1.06–1.91); *p* = 0.02) or low (OR = 1.51(1.08–2.11); *p* = 0.02) food security were significantly more likely to have food price concerns than participants experiencing high food security. However, surprisingly experiencing very low food security was not found to be associated with food price concerns. Food security status was also associated with food waste concerns (Regression 2). Participants experiencing marginal (OR = 0.74(0.56–0.97); *p* = 0.03), low (OR = 0.67(0.49–0.90); *p* = 0.01), and very low (OR = 0.66(0.48–0.92); *p* = 0.01) food security were all significantly less likely to have food waste concerns than participants experiencing high food security. Additionally, food security status appeared to be associated with food quality concerns (Regression 3), yet while participants experiencing very low food security were significantly less likely (OR = 0.65(0.47–0.90); *p* = 0.01) to be concerned regarding food quality than participants experiencing high food security, as indicated above, adding food security status as an independent variable did not significantly improve the fit of the model (Table 4; *p* = 0.08). No significant associations were found between food security status and concern about being able to eat healthily (Regression 4).

### Exploratory research questions (RQ2 and RQ3)

#### Descriptive statistics

For the exploratory research questions focused on food support, only participants identifying as food insecure (low or very low food security) were included in the analysis (*N* = 467; low food security *N* = 236 (50.5%), very low food security *N* = 231 (49.5%)). Hence, food security status was considered as a binary variable in these analyses.

The descriptive statistics of the characteristics of the reduced sample are provided in Supplementary Material 3. In terms of self-reported receipt of food support, larger proportions within this sample (compared with the overall survey sample) had experience of using a food bank (10.5%) or a social supermarket (8.6%). Participants in the very low food security group (food bank 18.6%; social supermarket 13.4%) showed higher levels of use than participants in the low food security group (food bank 2.5%; social supermarket 3.8%) for both food support types.

#### Logistic regressions 5 & 6: Characteristics of food support users (RQ2)

VIFs were calculated for the model in RQ2, with VIFs < 2 for each covariate, indicating minimal multicollinearity between covariates.^(49)^ Logistic regressions were conducted with food bank use (Regression 5) or social supermarket use (Regression 6) as the dependent variable and demographic characteristics as the independent variables. Independent variables were inputted simultaneously into the model. The overall model was statistically significant when both food bank use (Ⲭ^2^(12): 81.16, *p* < 0.001, McFadden *R* ^2^: 0.26, classification rate: 91.0%), and social supermarket use (Ⲭ^2^(12): 52.15, *p* < 0.001, McFadden *R* ^2^: 0.19, classification rate: 91.4%) were the dependent variable.

People experiencing very low food security or having a long-term health condition were significantly more likely to use a food bank (very low food security OR = 6.05(2.42–15.15), *p* < 0.001; long-term health condition OR = 3.91(1.77–8.63), *p* = 0.00) or a social supermarket (very low food security OR = 2.40(1.13–5.52), *p* = 0.02; long-term health condition OR = 3.17(1.49–7.00), *p* = 0.00) (See Supplementary Material 6). Additionally, people who were unemployed or not working were significantly more likely to use a food bank (OR = 2.60(1.15–5.90); *p* = 0.02), while people identifying as non-White were more likely to use a social supermarket (OR = 4.41(1.60–11.70); *p* = 0.01). All other associations were non-significant.

Sensitivity analysis was conducted with the whole sample, as opposed to the reduced sample. All associations remained significant. Other expected significant associations were also found including for total annual household income and between food security levels (see Supplementary Material 7).

#### Logistic regressions 7–14: Food support use and food-related concerns (RQ3)

Again, VIFs were calculated for each step in the two-step model. All VIFs < 2 for the covariates in the models in RQ3, indicating minimal multicollinearity between covariates.^(49)^ Upon adding food bank use (coded as ‘Yes’ = 1 and ‘No’ = 0) into the model, the model fit was only significantly improved for concerns about being able to eat healthily (*p* = 0.00) while for social supermarket use (again, coded as ‘Yes’ = 1 and ‘No’ = 0), the model fit was only significantly improved for food price concerns (0.01; see Table 5).

**Table 5.** Change in model fit statistics for regressions with food-related concerns against covariates and food support use

Food-related concern	ΔⲬ^2^	*Df*	*p*	Δ(McFadden *R* ^2^)	Δ(Classif-ication rate)	ΔAIC
*Food bank use*						
Regression 7: Food prices	1.16	1	0.28	0.00	0.0%	0.84
Regression 8: Food waste	0.29	1	0.59	0.00	−0.2%	1.71
Regression 9: Food quality	0.32	1	0.57	0.00	0.6%	1.68
Regression 10: Being able to eat healthily	9.44	1	***0.00***	0.01	1.5%	−7.43
*Social supermarket use*						
Regression 11: Food prices	6.29	1	***0.01***	0.01	1.7%	−4.29
Regression 12: Food waste	0.05	1	0.81	0.00	0.2%	1.94
Regression 13: Food quality	3.2	1	0.07	0.00	0.2%	−1.20
Regression 14: Being able to eat healthily	0.96	1	0.33	0.00	−0.4%	1.04

RQ2: **Dependent variable:** Food bank use or social supermarket use; **Covariates:** ethnicity, country, gender, age band, urban/rural classification, employment status, total annual household income, total household size, long-term health condition, food security status.

RQ3: **Dependent variable:** Food-related concern; **Independent variable:** Food bank use or social supermarket use; **Covariates:** ethnicity, country, gender, age band, urban/rural classification, employment status, total annual household income, total household size, long-term health condition.

For Regression 7: Food prices, the only covariate that was significant was age. All other covariates were non-significant.

For Regression 8: Food waste, the only covariates that were significant were gender, age, and total household income. All other covariates were non-significant.

For Regression 9: Food quality, the only covariate that was significant was age. All other covariates were non-significant.

For Regression 10: Being able to eat healthily, the only covariate that was significant was age. All other covariates were non-significant.

For Regression 11: Food prices, the only covariate that was significant was age. All other covariates were non-significant.

For Regression 12: Food waste, the only covariates that were significant were gender, age, and total household income. All other covariates were non-significant.

For Regression 13: Food quality, the only covariate that was significant was age. All other covariates were non-significant.

For Regression 14: Being able to eat healthily, the only covariate that was significant was age. All other covariates were non-significant.

**Figure 2.**
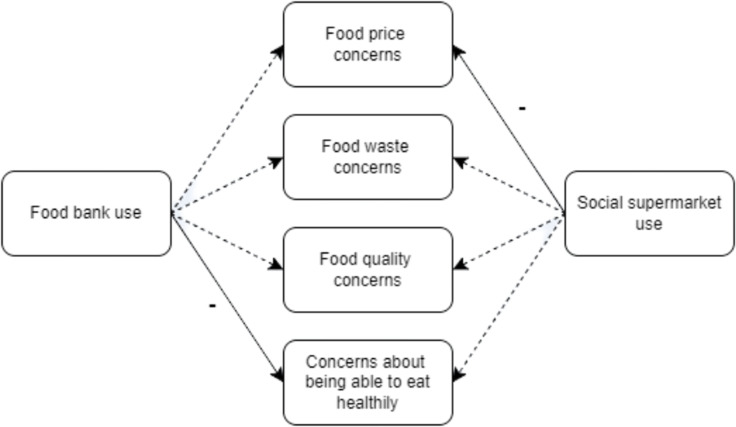
A visual overview of the associations between food bank use and social supermarket use with food-related concerns. Solid arrows denote significant associations and dashed arrows denote non-significant associations. Negative associations are represented by a ‘−’ above their associated arrow.

Food bank use was only associated with a lower likelihood of being concerned about being able to eat healthily (Regression 10: OR = 0.33(0.15–0.70); *p* = 0.00), while social supermarket use was only associated with a lower likelihood of having food price concerns (Regression 11: OR = 0.40(0.20–0.81); *p* = 0.01; see Supplementary Material 8 and Figure 2). All other associations were non-significant.

Sensitivity analysis was conducted with the whole sample, as opposed to the reduced sample. The association between food bank use and lower concerns about being able to eat healthily remained significant, while for social supermarket use, the association with food prices became non-significant, likely due inclusion of people experiencing marginal food insecurity using social supermarkets (see Supplementary Material 9 and 10).

## Discussion

Food and You 2 Wave 6 data were used to identify the associations between food security status and food-related concerns. Exploratory analyses were also conducted to identify the characteristics of participants using food banks or social supermarkets compared to non-users, and the associations between food bank or social supermarket use and food-related concerns. Experiencing marginal or low food security (relative to high food security) was associated with increased likelihood of having concerns about food prices, but surprisingly this association was not seen in participants experiencing very low food security. Additionally, food waste concerns were significantly less likely with decreasing level of food security. Experiencing very low food security or having a long-term health condition were both significantly associated with higher likelihood of reporting receipt of food support from a food bank or social supermarket. Being unemployed or not working was significantly associated with higher likelihood of food bank use, while identifying as non-White was significantly associated with higher likelihood of receiving support from a social supermarket. Finally, food bank use was associated with a lower likelihood of concerns about being able to eat healthily while social supermarket use was associated with a lower likelihood of having food price concerns in people experiencing more severe forms of food insecurity.

Collectively the findings highlight that food-related concerns differ across different levels of food security severity, most notably the experience of food price concerns. The higher food price concerns in persons experiencing marginal and low food security (relative to those experiencing high food security) are intuitive and support the hypothesis for RQ1. However, what is surprising is that, contrary to the hypothesis, this association was not present in those experiencing very low food security (i.e. high food insecurity). Our subsequent exploratory analyses indicated that participants of very low food security status were over twice as likely to use a social supermarket and just over six times more likely to use a food bank than participants of low food security status. In turn, attending a social supermarket was associated with lower likelihood of food price concerns, whereas food bank use was not. Taken together, these findings tentatively suggest that for households in very low food security, social supermarkets may reduce worry about affording sufficient food, which has been linked to worse mental health outcomes previously,^(24)^ and thus might offer a potentially more psychologically supportive alternative to more traditional food banks.

Previous research indicates that Black, Asian, and minority ethnic (BAME) groups are often deterred from attending food banks due to a lack of culturally appropriate foods and affiliations to religious organisations.^(12,31,38,50)^ In contrast, social supermarkets have been suggested as offering a more dignified alternative to traditional food bank models as they allow choice and respect individual food needs and cultural preferences.^(42)^ In the present study, being of non-White ethnicity was predictive of social supermarket use but not food bank use, providing initial evidence to suggest that social supermarkets may act as a more culturally sensitive approach to food support compared to food banks. Therefore, social supermarkets may also be a positive step in improving the effectiveness of the support received from food support services for some marginalised groups. However, within the small number of studies conducted around this topic, concerns persist over the ability of households to access a stable and healthy diet in a completely dignified way using social supermarkets alone.^(42,51)^ Therefore, progress is still required in working towards an alternative and more effective long-term solution.

Yet, while social supermarkets may offer some protection from food price concerns, especially for people in very low food security, our findings highlight an elevated level of food price concerns in people experiencing low and marginal food security (relative to people experiencing high food security), coupled with a lower likelihood of using social supermarkets compared to people experiencing very low food security (See Supplementary Material 7). Subsequently, this suggests that people in low and marginal food security may be less likely to benefit from the protective effect that social supermarkets may provide against food price concerns. Often, research focuses on the experiences of people most severely affected by food insecurity, however these findings suggest that those occupying the space between food security and more severe forms of food insecurity may be missed in terms of adequate support and highlights the importance of developing interventions which target the reduction of food price concerns for those in marginal and low food security. Research commonly demonstrates the effortful implementation of coping strategies such as strict budgeting, resourcefulness and buying food according to supermarket offers in households experiencing less severe forms of food insecurity to enable the purchasing of healthy foods and to mitigate against the need to access food support.^( 3,30,52,53)^ In a study by Stone, Christiansen^(54)^ participants with varying levels of food insecurity reported that supermarket interventions based on price discounts, offers, and promotions would be most helpful in supporting the purchasing of healthy foods. Therefore, with healthy foods now costing twice as much per calorie than less healthy foods,^(34,35)^ engaging supermarkets in interventions to reduce the price of healthy foods through such methods of incentivisation may enable the easier implementation of coping strategies and reduction of food price concerns in people in low and marginal food insecurity, in turn decreasing psychological distress and increasing capacity to make more healthy food choices.^(16,53)^

Meanwhile, contrary to the hypothesis, decreasing level of food security (i.e. *increasing* food *in*security) was associated with decreased likelihood of food waste concerns. One explanation for this may be that households experiencing lower levels of food security live in food deficit and possess less freedom to waste food, already implementing increasingly meticulous food management strategies and resourcefulness to minimise waste with increasing food insecurity severity.^(3,28,30)^ Therefore, there may be minimal food waste opportunities in such households and therefore wasting food is of less concern. Meanwhile households experiencing high food security may represent the ‘worried well’, being in a position in which their ability to afford sufficient (and/or surplus) food grants a certain privilege of worry and concern over waste due to more food waste opportunities. Alternatively, research suggests that diet quality, including inclusion of fresh foods, decreases as food security status decreases.^(20,55,56)^ Reasons for this may include the relative unaffordability of healthy, fresh foods such as fruit and vegetables compared to less healthy, more processed foods, and a lack of availability of, and access to, fresh foods in some deprived areas.^(34,57,58)^ It is therefore possible that people with a lower food security status are less concerned about food waste, as they purchase and consume less fresh produce but more processed and non-perishable foods that do not spoil or warrant wasting.

Furthermore, exploratory analysis found that food bank use was associated with decreased likelihood of concerns about being able to eat healthily. As food banks are often unable to provide a balanced and nutritious diet,^(59,60)^ it is possible that the participants reporting food bank use in the survey were generally less concerned about health, or possessed an ‘eating to survive’ mindset^(16)^ as a result of their food insecurity, in which accessing food of any kind is valued, despite its nutritional quality. However, previous evidence suggests individuals using food banks place value on eating healthily,^(12,16,61)^ reinforcing the substantial lack of agency one experiences when accessing a food bank.^(62)^.

## Strengths and limitations

To our knowledge, no other research has mobilised the Food and You 2 dataset to explore food-related concerns, particularly in relation to food insecurity and food support use. Moreover, there is little other research which quantitatively explores food-related concerns including food prices, waste, quality, and being able to eat healthily through other means and in other contexts. Further strengths of this study lie in the scope and reach of the Food and You 2 survey, which is a nationally representative survey with a large sample size. Food and You 2 also uses the USDA 10-item AFSSM,^(8)^ which is validated and widely used to measure food security status across high income countries, in particular the UK. Additionally, digital poverty, defined as a lack of access to the ‘online world’^(63,64)^ is a common issue in low-income households that may also be experiencing other inequalities such as food insecurity.^(64,65)^ Research indicates that 45% of UK households with children do not have the adequate access, skills or equipment to partake in modern online life.^(63)^ This can create sampling bias in research by limiting lower income households from participating. However, this is mitigated within the Food and You 2 survey through the option to complete either online or via a postal version, with the differences between versions being accounted for within the analysis.^(43)^

On the other hand, there are limitations that should be considered. First, Food and You 2 represents a cross-sectional analysis, through which determination of causality of associations is not possible. Further limitations lie in the self-report nature of the survey which may lead to respondents providing what they perceive as socially desirable responses, manifesting bias. For example, within the present study, only 2.6% of respondents reported using a food bank and 2.4% reported using a social supermarket, despite 10% of respondents being classified as experiencing very low food security. This could be due to underreporting of food support use because of the shame and stigma that surround receiving food support within society.^(62,66)^ Similarly, food security levels may have also been underreported by respondents. The sample size of the reduced sample, made up of participants experiencing low or very low food security was also small in comparison to the overall survey sample, which may have affected the statistical analysis (although sensitivity analysis suggests otherwise). Further exploration of the characteristics of food support users and the association between food support use and food-related concerns across a larger sample would be of use in further validating the findings. Additionally, some respondents may find some of the variables ambiguous. For example, within the food-related concerns variable, food quality may relate to nutritional quality, actual quality, or brand prestige. Therefore, answering ‘Yes’ to having food quality concerns may not have necessarily just referred to being concerned about being able to eat food of a ‘safe and nutritious’ quality as per the food insecurity definition. Similarly, respondents may have been concerned about food waste for environmental reasons, not necessarily for financial reasons. This may have led to overreporting of concerns within respondents experiencing high food security.

## Conclusion

Overall, the findings highlight the importance of exploring the differential experience of food-related concerns across food insecurity severity, indicating that food price concerns may be most salient for people experiencing marginal and low food security, but surprisingly less salient for people experiencing very low food security. Meanwhile, food waste concerns appeared to decrease with decreasing food security. Factors found to predict food support use included experiencing very low food security, having a long-term health condition, being unemployed or not working and identifying as non-White. Food bank use was associated with a lower likelihood of concerns about being able to eat healthily, while social supermarket use was associated with lower likelihood of food price concerns. The findings suggest a potentially important role of social supermarkets in helping to address food price concerns for people experiencing very low food security. They also highlight the need for ongoing support for people experiencing marginal and low food security to address concerns about food prices in these groups and to ultimately promote health and wellbeing.

## Supporting information

Taylor et al. supplementary material Taylor et al. supplementary material

## Supplementary material

The supplementary material for this article can be found at https://doi.org/10.1017/jns.2025.10065
