# Supplementary material for: Associations between food-related concerns, food security status, and food support use: a secondary analysis of the Food and You 2: Wave 6 dataset
Source: J Nutr Sci. 2026 Jan 9;15:e8. doi: 10.1017/jns.2025.10065 (PMC12800539; doi:10.1017/jns.2025.10065)
Supplement: Taylor et al. supplementary material [file S2048679025100657sup001.docx]

Tetrachoric correlation matrix for food-related concern variables

|  | | Food-related concern | | | |
| --- | --- | --- | --- | --- | --- |
|  |  | Food prices | Food waste | Food quality | Being able to eat healthily |
| Food-related concern | Food prices | 1.00 | 0.27 | 0.28 | 0.28 |
|  | Food waste | 0.27 | 1.00 | 0.35 | 0.41 |
|  | Food quality | 0.28 | 0.35 | 1.00 | 0.51 |
|  | Being able to eat healthily | 0.28 | 0.41 | 0.51 | 1.00 |

*Model fit statistics for regressions 1-4 with food-related concerns and covariates only*

| *Food-related concern* | *Ⲭ^2^* | *Df* | *p* | *McFadden R^2^* | *Classification rate* |
| --- | --- | --- | --- | --- | --- |
| Food price | 33.09 | 9 | ***<0.001*** | 0.01 | 32.8% |
| Food waste | 142.90 | 9 | ***<0.001*** | 0.05 | 66.3% |
| Food quality | 72.69 | 9 | ***<0.001*** | 0.02 | 64.3% |
| Being able to eat healthily | 60.57 | 9 | ***<0.001*** | 0.02 | 55.4% |

*Descriptive statistics of the reduced sample characteristics (RQ2 and RQ3; N=467)*

| *Measure* | *Full sample (N=467)*  *n(%)* | *Low food security*  *(N=236)*  *n(%)* | | *Very low food security (N=231)*  *n(%)* | |
| --- | --- | --- | --- | --- | --- |
| Ethnicity |  | |  | |  |
| White | 428 (91.6) | | 214(90.7) | | 214(92.6) |
| Other ethnic group | 39 (8.4) | | 22(9.3) | | 17(7.4) |
| Country |  | |  | |  |
| England | 200 (42.8) | | 103(43.6) | | 97(42.0) |
| Wales | 121 (25.9) | | 60(25.4) | | 61(26.4) |
| Northern Ireland | 146 (31.3) | | 73(30.9) | | 73(31.6) |
| Gender |  | |  | |  |
| Male | 161 (34.5) | | 86(36.4) | | 75(32.5) |
| Female | 306 (65.5) | | 150(63.6) | | 156(67.5) |
| Age band |  | |  | |  |
| 16-24 | 33 (7.1) | | 17(7.2) | | 16(6.9) |
| 25-34 | 128 (27.4) | | 53(22.5) | | 75(32.5) |
| 35-44 | 130 (27.8) | | 58(24.6) | | 72(31.2) |
| 45-54 | 71 (15.2) | | 39(16.5) | | 32(13.9) |
| 55-64 | 58 (12.4) | | 39(16.5) | | 19(8.2) |
| 65-79 | 44 (9.4) | | 28(11.9) | | 16(6.9) |
| 80+ | 3 (0.6) | | 2(0.8) | | 1(0.4) |
| Urban/rural classification |  | |  | |  |
| Urban | 346 (74.1) | | 172(72.9) | | 174(75.3) |
| Rural | 121 (25.9) | | 64(27.1) | | 57(24.7) |
| Employment status |  | |  | |  |
| Working | 313 (67.0) | | 165(69.9) | | 148(64.1) |
| Unemployed/not working | 109 (23.3) | | 38(16.1) | | 71(30.7) |
| Retired | 45 (9.6) | | 33(14.0) | | 12(5.2) |
| Total annual household income |  | |  | |  |
| < £19,000 | 212 (45.4) | | 89(37.7) | | 123(53.2) |
| £19,000-£31,999 | 146 (31.3) | | 75(31.8) | | 71(30.7) |
| £32,000-£63,999 | 92 (19.7) | | 61(25.8) | | 31(13.4) |
| £64,000-£95,999 | 13 (2.8) | | 7(3.0) | | 6(2.6) |
| > £96,000 | 4 (0.9) | | 4(1.7) | | 0(0) |
| Total household size |  | |  | |  |
| 1 | 63 (13.5) | | 32(13.6) | | 31(13.4) |
| 2 | 137 (29.3) | | 83(35.2) | | 54(23.4) |
| 3 | 98 (21.0) | | 44(18.6) | | 54(23.4) |
| 4 | 100 (21.4) | | 45(19.1) | | 55(23.8) |
| 5+ | 69 (14.8) | | 32(13.6) | | 37(16.0) |
| Long-term health condition |  | |  | |  |
| Has a long-term health condition | 197 (42.2) | | 83(35.2) | | 114(49.4) |
| Doesn't have a long-term health condition | 270 (57.8) | | 153(64.8) | | 117(50.6) |
| Food bank use |  | |  | |  |
| Yes | 49 (10.5) | | 6(2.5) | | 43(18.6) |
| No | 418 (89.5) | | 230(97.5) | | 188(81.4) |
| Social supermarket use |  | |  | |  |
| Yes | 40 (8.6) | | 9(3.8) | | 31(13.4) |
| No | 427 (91.4) | | 227(96.2) | | 200(86.6) |

*Model fit statistics for regressions 7-14 with food-related concerns and covariates only (using the reduced sample of participants identifying as food insecure (low or very low food security))*

| *Food-related concern* | *Ⲭ^2^* | *Df* | *p* | *McFadden R^2^* | *Classification rate* |
| --- | --- | --- | --- | --- | --- |
| Food price | 22.99 | 9 | ***0.006*** | 0.04 | 71.3% |
| Food waste | 25.00 | 9 | ***0.003*** | 0.04 | 59.3% |
| Food quality | 31.13 | 9 | ***<0.001*** | 0.05 | 59.1% |
| Being able to eat healthily | 17.74 | 9 | ***0.038*** | 0.03 | 62.5% |

Associations between food security status and food-related concerns (RQ1).

|  |  | *Food-related concern*  Odds ratio (95% CI) | | | | | | |
| --- | --- | --- | --- | --- | --- | --- | --- | --- |
|  |  | Regression 1: Food prices | | Regression 2: Food waste | | Regression 3: Food quality | | Regression 4: Being able to eat healthily |
| *Food security status* | Marginal | **1.43* (1.06-1.91)** | **0.74* (0.56-0.97)** | | 0.92 (0.70-1.21) | | 1.10 (0.85-1.44) | |
|  | Low | **1.51* (1.08-2.11)** | **0.67* (0.49-0.90)** | | 0.90 (0.67-1.23) | | 0.97 (0.72-1.31) | |
|  | Very low | 1.12 (0.79-1.57) | **0.66* (0.48-0.92)** | | **0.65* (0.47-0.90)** | | 0.76 (0.54-1.05) | |

High food security status was used as the reference category for all logistic regressions in the table.

p<0.05*, p<.001**

Associations between food support use and demographic covariates (RQ2)

|  |  | *Type of food support*  Odds ratio (95% CI) | |
| --- | --- | --- | --- |
|  |  | Regression 5: Food bank use | Regression 6: social supermarket use |
| *Predictor* | Food security status | **6.05** (2.42-15.15)** | **2.40* (1.13-5.52)** |
|  | Gender | 0.79 (0.37-1.67) | 0.98 (0.46-2.18) |
|  | Age | 0.90 (0.67-1.22) | 0.78 (0.57-1.04) |
|  | Total household size | 1.29 (0.96-1.74) | 1.12 (0.83-1.51) |
|  | Long-term health condition | **3.91* (1.77-8.63)** | **3.17* (1.49-7.00)** |
|  | Urban/rural classification | 1.91 (0.87-4.19) | 1.57 (0.68-3.49) |
|  | Country(Wales)^∆^ | 1.40 (0.61-3.21) | 0.74 (0.29-1.76) |
|  | Country(Northern Ireland)^∆^ | 0.82 (0.35-1.88) | 0.85 (0.36-1.91) |
|  | Employment status(Unemployed/not working)^+^ | **2.60* (1.15-5.90)** | 1.40 (0.62-3.16) |
|  | Employment status(Retired)^+^ | 0.61 (0.10-3.87) | 0.23 (0.00-2.35) |
|  | Ethnicity | 1.40 (0.38-5.11) | **4.41* (1.60 -11.70)** |
|  | Total annual household income | 0.54 (0.29-1.00) | 0.64 (0.35-1.09) |

P<0.05*, p<0.001**

^∆^With Country(England) as a reference category. Further analysis to explore associations between Wales and Northern Ireland were conducted, replacing the reference category with Country(Wales), yet no significant associations were found for food bank use (OR=0.58, 95% CI=0.24-1.44, p=0.242) or social supermarket use (OR=1.14, 95% CI=0.43-3.11, p=0.796).

^+^With Employment status(Working) as a reference category. Further analysis to explore associations between Unemployed/not working and Retired were conducted, replacing the reference category with Employment status(Unemployed/not working), yet no significant associations were found for food bank use (OR=0.23, 95% CI=0.04-1.46, p=0.120) or social supermarket use (OR=0.17, 95% CI=0.0-1.68, p=0.148).

Due to problematic confidence intervals (i.e. Employment status(Retired) 95% CI=0.00-inf) a penalised likelihood regression (Firth, 1993) was used for the models relating to social supermarket use.

For reference, low food security status, male gender, not having a long-term health condition, urban classification, and White ethnicity were used as the reference categories for the binary variables. Age, total household size and total annual household income were treated as continuous variables within this analysis.

Sensitivity analysis: Associations between food support use and demographic characteristics (RQ2)

|  |  | *Type of food support*  Odds ratio (95% CI) | |
| --- | --- | --- | --- |
|  |  | Regression 5: Food bank use | Regression 6: social supermarket use |
| *Predictor* | Food security status(Marginal)^ꓳ^ | **5.60*(1.57-19.98)** | **3.27*(1.16-9.22)** |
|  | Food security status(Low) ^ꓳ^ | **3.93*(1.02-15.20)** | **2.93*(1.01-8.53)** |
|  | Food security status(Very low) ^ꓳ^ | **23.75**(7.44-75.83)** | **7.97**(3.06-20.74)** |
|  | Gender | 0.75(0.40-1.39) | 1.27(0.67-2.39) |
|  | Age | 0.89(0.69-1.15) | 0.84(0.65-1.07) |
|  | Total household size | 1.18(0.91-1.52) | 1.27(0.98-1.64) |
|  | Long-term health condition | **2.55*(1.33-4.91)** | **3.47**(1.84-6.57)** |
|  | Urban/rural classification | 1.64(0.84-3.20) | 1.12(0.56-2.25) |
|  | Country(Wales)^∆^ | 1.12(0.55-2.28) | 0.50(0.23-1.09) |
|  | Country(Northern Ireland)^∆^ | 0.81(0.39-1.67) | 0.68(0.34-1.37) |
|  | Employment status(Unemployed/not working)^+^ | **2.14*(1.07-4.25)** | 1.38(0.70-2.71) |
|  | Employment status(Retired)^+^ | 0.58(0.15-2.17) | 0.47(0.11-1.90) |
|  | Ethnicity | 1.67(0.60-4.68) | **2.96*(1.25-7.02)** |
|  | Total annual household income | **0.52*(0.33-0.82)** | **0.67*(0.46-0.98)** |
|  |  |  |  |

p<0.05*, p<0.001**

^ꓳ^With Food security status(High) as a reference category. Further analysis to explore associations between (i) Marginal and Low food security, and (ii) Marginal and Very low food security were conducted, replacing the reference category with Food security status(Marginal). No significant association was found between (i) Marginal and Low food security for food bank use (OR=0.70, 95% CI=0.22-2.19, p=0.543) or social supermarket use (OR=0.90, 95% CI=0.33-2.45, p=0.831). A significant association was found between (ii) Marginal and Very low food security for both food bank use (OR=4.24, 95% CI=1.75-10.27, p=0.001) and social supermarket use (OR=2.44, 95% CI=1.03-5.79, p=0.043). Further analysis to explore associations between (iii) Low and Very low food security was also conducted, replacing the reference category with Food security status(Low). A significant association was found between (ii) Low and Very low food security for both food bank use (OR=6.04, 95% CI=2.45-14.90, p<0.001) and social supermarket use (OR=2.72, 95% CI=1.22-6.07, p=0.014).

^∆^With Country(England) as a reference category. Further analysis to explore associations between Wales and Northern Ireland were conducted, replacing the reference category with Country(Wales), yet no significant associations were found for food bank use (OR=0.72, 95% CI=0.33-1.57, p=0.409) or social supermarket use (OR=1.35, 95% CI=0.57-3.19, p=0.498).

^+^With Employment status(Working) as a reference category. Further analysis to explore associations between Unemployed/not working and Retired were conducted, replacing the reference category with Employment status(Unemployed/not working), yet no significant associations were found for food bank use (OR=0.27, 95% CI=0.07-1.02, p=0.054) or social supermarket use (OR=0.34, 95% CI=0.08-1.44, p=0.142).

For reference, male gender, not having a long-term health condition, urban classification, and White ethnicity were used as the reference categories for the binary variables. Age, total household size and total annual household income were treated as continuous variables within this analysis.

Associations between food support use and food-related concerns (RQ3)

|  |  | *Outcomes: Types of concern*  Odds ratios (95% CI) | | | |
| --- | --- | --- | --- | --- | --- |
|  |  | Food prices | Food waste | Food quality | Being able to eat healthily |
| *Type of food support* | Food bank use | Regression 7  0.69 (0.35-1.35) | Regression 8  1.19 (0.63-2.26) | Regression 9  0.83 (0.44-1.58) | Regression 10  **0.33* (0.15-0.70)** |
|  | Social supermarket use | Regression 11  **0.40* (0.20-0.81)** | Regression 12  0.92 (0.46-1.85) | Regression 13  0.53 (0.26-1.07) | Regression 14  0.69 (0.33-1.45) |

P<0.05*, p<0.001**

*Sensitivity analysis: Change in model fit statistics for regressions with food-related concerns against covariates and food bank use (RQ3).*

| *Food-related concern* | *∆Ⲭ^2^* | *Df* | *p* | *∆(McFadden*  *R^2^)* | | *∆(Classif-ication rate)* | *∆AIC* |
| --- | --- | --- | --- | --- | --- | --- | --- |
| *Food bank use* | | | | | | | |
| Food prices | 1.33 | 1 | 0.248 | | 0.00 | 34.39 | 0.67 |
| Food waste | 0.01 | 1 | 0.962 | | 0.00 | 0.00 | 2.00 |
| Food quality | 2.64 | 1 | 0.105 | | 0.00 | 0.30 | -0.64 |
| Being able to eat healthily | 10.34 | 1 | ***0.001*** | | 0.00 | 0.69 | 8.34 |
| *Social supermarket use* | | | | | | | |
| Food price concerns | 2.11 | 1 | 0.146 | | 0.00 | 34.39 | -0.11 |
| Food waste concerns | 0.01 | 1 | 0.937 | | 0.00 | 0.04 | 1.99 |
| Food quality concerns | 3.73 | 1 | 0.053 | | 0.01 | 0.22 | -1.73 |
| Being able to eat healthily | 0.75 | 1 | 0.386 | | 0.00 | 0.39 | 1.25 |

*For Food bank use: Food prices, the only covariates that were significant were age, total household size, country, employment status and total household income. All other covariates were non-significant.
For Food bank use: Food waste, the only covariates that were significant were gender, age, total household size and total household income. All other covariates were non-significant.
For Food bank use: Food quality, the only covariates that were significant were age, presence of a long-term health condition and total household income. All other covariates were non-significant.
For Food bank use: Being able to eat healthily, the only covariates that were significant were age, presence of a long-term health condition and total household income. All other covariates were non-significant.*

*For social supermarket use: Food prices, the only covariates that were significant were, age, total household size, urban/rural classification, country, employment status and total household income. All other covariates were non-significant.
For social supermarket use: Food waste, the only covariates that were significant were, gender, age, total household size and total household income. All other covariates were non-significant.
For social supermarket use: Food quality, the only covariates that were significant were, age, presence of a long-term health condition and total household income. All other covariates were non-significant.
For social supermarket use: Being able to eat healthily, the only covariates that were significant were age and total household income. All other covariates were non-significant.*

Sensitivity analysis: Associations between food support use and food-related concerns (RQ3)

|  |  | *Outcomes: Types of concern*  Odds ratios (95% CI) | | | |
| --- | --- | --- | --- | --- | --- |
|  |  | Food prices | Food waste | Food quality | Being able to eat healthily |
| *Type of food support* | Food bank use | 0.72(0.41-1.25) | 1.01(0.59-1.75) | 0.64(0.37-1.10) | **0.38(0.20-0.71)** |
|  | Social supermarket use | 0.65(0.37-1.15) | 0.98(0.56-1.72) | 0.58(0.33-1.01) | 0.78(0.44-1.38) |

P<0.05*, p<0.001**
